# Research on the Bioactivity of Plant Essential Oils on Armyworm [*Mythimna separata* (Walker)] Larvae

**DOI:** 10.3389/fchem.2022.936873

**Published:** 2022-06-29

**Authors:** Tao Wang, Yanling Ren, Jinyu Zhao, Yao Liu, Bin Xu, Maofa Yang, Wanling Zhao, Xinian Zheng, Juan Wang, Liuqiong Deng

**Affiliations:** ^1^ Guizhou Light Industry Technical College, Guiyang, China; ^2^ The Provincial Key Laboratory for Agricultural Pest Management in the Mountainous Region, Institute of Entomology, Guizhou University, Guiyang, China; ^3^ Guizhou Sino Grain Quality Supervision Center, Guiyang, China

**Keywords:** plant essential oil, antifeedant activity, repellent activity, fumigation activity, contact activity, synergistic effect, armyworm larvae

## Abstract

In order to find out the biological activity of plant essential oils on armyworm [*Mythimna separata* (Walker, 1865)] larvae and provide a theoretical basis for the biological control of armyworms, in this study, the antifeedant activity, repellent activity, fumigation activity, contact activity, and synergistic effect on indoxacarb of nine kinds of plant essential oils on armyworm larvae were determined. The results showed that lavender and citronella essential oils had the greatest impact on the antifeedant activity on armyworm larvae, and the antifeedant rate reached 100.00%. Meanwhile, rosemary essential oil revealed the best repellent activity on armyworm larvae with an average dwell time of 0 s at the content of 0.2%. Moreover, tea tree essential oil and lemon essential oil at the content of 2.0% had the best fumigation and contact activity against armyworm larvae, and the corrected mortality rates at 120 h were 86.67 and 66.67%, respectively. In addition, the combination of citronella essential oil and indoxacarb with the ratio of 5:1 had the best synergistic effect on armyworm larvae at 96 h, and the synergistic ratio was reached 100.00%. These findings will guide the development of new insecticides for controlling armyworm larvae.

## Introduction

The armyworm, *Mythimna separata* (Walker, 1865) (*Lepidoptera*: *Noctuidae*), also known as marching worms and shaving worms. It is an important migratory agricultural pest and is widely distributed worldwide (Liao et al., 2020) and hosted on grain crops, such as wheat, rice, cotton, millet, vegetables, and corn, as well as more than 104 species of plants in 16 families (Jiang et al., 2014). The armyworm has the characteristics of flocking, migratory, omnivorous, gluttonous, intermittent outbreaks, etc., has strong adaptability, prefers high-temperature and high-humidity environment, and has four to five large-scale migration processes every year. The armyworm larvae could eat up all the leaves of crops, often causing serious losses in a short period of time (Kong et al., 2019). In recent years, the insect has repeatedly appeared in high-density and concentrated damage in the northern country, posing a serious threat to food production (Duan et al., 2018). At present, the field control of armyworms is still dominated by traditional pesticides. However, the long-term application of traditional pesticides is not only destroys the ecological environment and affects the survival of natural enemies, but also significantly increases the drug resistance of armyworms to reduce the control effect (Wei et al., 2010; Dong et al., 2014; Zhao et al., 2017; Zhang et al., 2019; Liao et al., 2020). Therefore, it is of great significance to use multiple ways to coordinately regulate armyworm populations.

Plant essential oils are composed of a complex mixture of secondary metabolites, which have the advantages of being widely used, having low residues in the environment, not polluting crops, and not easy for pests to develop drug resistance. Their bioactivity research on insects has attracted increasing attention for many scholars (Sombra et al., 2020; Zhao et al., 2020; Zhang et al., 2022). The exogenous application of plant essential oils is often used for insecticidal, repellent, and antifeedant activity (Isman, 2015), for example, the repelling effect of mulberry leaf’s essential oil on the red grain thief (Xu et al., 2019), the contact and repelling activity of 11 kinds of plants’ essential oils on the tea orange gall mites (Li et al., 2019), the biological activity of lavender essential oil on *Spodoptera litura* (Zhang Z. et al., 2019), the repellent activity of lemon essential oil on the pink beetle *Tribolium castaneum* (Olivero-Verbel et al., 2010), and the repellent effect of citronella essential oil on the adults of *Aedes aegypti* (*Diptera*: *Culicidae*) (Suwansirisilp et al., 2013). In addition, some researchers have also paid attention to the research of plant essential oils on insect feeding, egg hatching, and pesticide synergy. For example, Zhang Y. et al. (2019) found that calamus essential oil had inhibitory effects on the feeding and egg hatching behavior of 2-day-old larvae of diamondback moth; Yang et al. (2021) showed that aloe vera essential oil had a good synergistic effect on the control of *Spodoptera frugiperda* by indoxacarb; An et al. (2012) showed that the combination of lemongrass oil and natural pyrethrin could improve its insecticidal effect; Wang et al. (2019) found that the plant’s essential oil D-limonene could increase the efficacy of imidacloprid in controlling *Gossypium gossypii*. To sum up, plant essential oils have become a hot spot in integrated pest control, which is of great significance to the development of new pesticides for armyworm; however, only few authors have reported the relevant studies on the biological activity of armyworms (Passreiter et al., 2005; Ma et al., 2010; Cárdenas-Ortega et al., 2015; Song et al., 2020).

## Materials and Methods

### Test Insects

The test armyworms were collected from wild corn fields in Guiyang City (26.387282°N, 106.625232°E), Guizhou Province, and raised to the 6th generation in the laboratory. Larvae are reared in plastic trays with 4–6 cm thick substrates and fresh and pesticide-free corn leaves, and then placed in an artificial climate box with the rearing temperature, relative humidity, and photoperiod of 24°C, 70 ± 5%, and 14L:10D, respectively. When the larvae reached 4-days-old, the well-developed armyworm larvae of basically the same size were selected for the experiments.

### Test Materials

Basil essential oil (content ≥90%), peppermint essential oil (content ≥97%), lemon essential oil (content ≥80%), rosemary essential oil (content ≥99%), citronella essential oil (content ≥85%), frankincense essential oil (content ≥85%), eucalyptus essential oil (content ≥95%), tea tree essential oil (content ≥95%), and lavender essential oil (content ≥85%), which were proved to have good insecticidal activity (Yu et al., 2018; Sombra et al., 2020), were purchased from Guangdong Biotechnology Co., Ltd. (Guangdong, China). Indoxacarb EC (150 g/L) was purchased from FMC Corporation (Henan, China).

### Feeding Behavior Test on Armyworm Larvae

Each plant’s essential oil was divided into three content gradients of 2.0, 0.5, and 0.2%, respectively, with acetone according to the reported method by Sombra et al. (2020). Fresh corn leaves (5 g) with 10 μL of the test solutions of the plant’s essential oils on both sides were put into a self-made, transparent, and odor-free plastic box (length 24.5 cm, width 20.5 cm, and height 6.3 cm). Acetone served as a negative control. Then, 15 4-day-old armyworm larvae, which were pre-starvation treated for 4 h, were inserted into each self-made insect box. Each treatment was repeated three times. After 24 h treatment, the antifeedant rate and mortality rate were calculated according to the calculation method of Li et al. (2014).

Antifeedant rate (%) = (Feeding quality of control group−Feeding quality of treatment group)/Feeding quality of control group × 100.

Mortality rate (%) = Number of dead armyworm larvae/Number of test armyworm larvae × 100.

### Behavioral Response Test on Armyworm Larvae

The behavioral response test of 9 plant essential oils on armyworm larvae was tested according to the research method of Guarino et al. (2021). Each plant essential oil (50 μL) with the content of 2.0, 0.5, and 0.2%, respectively., was dropped in a 3 × 4 cm filter paper strip, then place the filter paper in one of the gas sources bottles, while the control gas source bottle was added with the same amount of acetone. To reduce experimental error, the filter paper was changed every 5 worms, and each treatment was repeated 30 times. The temperature of the test room was controlled at 28 ± 1°C by an air conditioner. An air pump (Aco-5505, Guangdong Haili Group Co., Ltd., all instruments are connected by rubber pipes with inner and outer diameters of 6 and 9 mm, respectively) was used to generate airflow. Then the airflow was passed through 1000 ml bottles which were filled with activated carbon to purify impurities, then passed through a 250 ml humidity bottle containing ultrapure water to increase air humidity. The airflow rate was adjusted through the glass rotor tachometer (flow rate 500 ml/min), then the airflow passed through the 250 ml gas source bottle to carry the smell and finally entered the Y-shaped pipe (stem 20 cm; arms 15 cm at 140 angles; stem internal diameter 5 cm, arms internal diameter 3.5 cm, place in an evenly lit area). After 5 min of ventilation, the armyworm larvae were placed in the middle of the main stem (3–5 cm away from the Y-pipe connection) and continued to observe for 300 s. The time when the armyworm larvae entered the experimental group (test arm) and the control group (the armyworm larvae entered the blank arm and the stem 5 cm away from the connection) was recorded. The dwell time is calculated by the following formula.

Average dwell time (s) = Test or control armyworm larvae active time/ (Number of armyworm larvae of treatment group + Number of armyworm larvae of the control group).

### Fumigation Activity on Armyworm Larvae

The fumigation activity of nine plant essential oils on armyworm larvae was tested according to the research method of Wu et al. (2015). Each plant essential oil (15 μL), with the contents of 2.0, 0.5, and 0.2%, respectively, was dripped in a rectangular filter paper (1.5 cm × 4 cm), then hang the filter paper vertically on the middle of a 1 L bottle which contained 30 4-day-old armyworm larvae inside. Acetone served as a negative control. Each treatment was repeated five times. After 24, 48, 72, 96, and 120 h of treatment, the corrected mortality rate is calculated using Abbott’s formula (Zhang Z. et al., 2019).

Corrected mortality rate (%) = (mortality rate of treatment group−mortality rate of the control group)/(1−mortality rate of the control group) × 100.

### Contact Activity on Armyworm Larvae

The contact activity of nine plant essential oils on armyworm larvae was tested according to the research method of Yuan et al. (2018). Three content gradients (2.0, 0.5, and 0.2%) of each plant's essential oil were placed on the prothorax and back of the armyworm with a micro-dropper, respectively. Then, 15 4-day-old armyworm larvae were carefully transferred to the Petri dish. Acetone served as a negative control. Three replicates were conducted for each treatment. After 24, 48, 72, and 96 h of treatment, the corrected mortality rate is determined using Abbott’s formula.

### Synergistic Ratio on Armyworm Larvae

The synergistic effects of plant essential oils and indoxacarb on armyworm larvae were determined according to the reported method by Xiong et al. (2019). The indoxacarb EC was diluted to the content of 0.025% with the purified water and mixed with different contents of plant essential oils with the ratio of 5:1, 10:1, and 20:1, respectively. Fifteen test armyworm larvae were placed in an insect breeding box (diameter 25 cm, height 8 cm) which was lined with filter paper. Each treatment was repeated 3 times. Then, the insect breeding box was placed under the potter spray tower for quantitative spray treatment (2 ml, pressure 103.4 kPa, settling time 30 s) and placed in an industrial climate box at 25°C with the photoperiod of 14L:10D. After 24, 48, 72, and 96 h of treatment, the corrected mortality rate is determined using Abbott’s formula, and the synergistic ratios are determined by the following formula.

Synergistic ratio (%) = corrected mortality rate of the combination of plant essential oils and indoxacarb)/corrected mortality rate of indoxacarb × 100.

### Statistical Analysis

All data in this study were analyzed using SPSS version 23.0 software (SPSS Inc., Chicago, United States). Duncan’s new complex extreme difference method was used to test the different significance of the data, *p* values below 0.05 were considered significant differences.

## Result

### Feeding Behavior Test on Armyworm Larvae

Table 1 showed that the three content gradients (2.0, 0.5, and 0.2%) of lavender essential oil and citronella essential oil had the greatest impact on the armyworm larvae, and antifeedant rate and mortality rate are 100.00%. The antifeedant rate and mortality rate of basil essential oil, peppermint essential oil, and rosemary essential oil also reached 100% at the content levels of 2.0 and 0.5%.

**TABLE 1:** The antifeedant rate and mortality rate of nine plant essential oils on armyworm larvae.

Plant essential oils	Content (%)	Antifeedant rate (%)*	Mortality rate (%)*
Basil essential oil	2.0	100.00 ± 0.00A	100.00 ± 0.00A
Basil essential oil	0.5	96.20 ± 3.80A	98.60 ± 1.40A
Basil essential oil	0.2	59.40 ± 3.67C	78.80 ± 4.95B
Frankincense essential oil	2.0	21.60 ± 2.98E	1.40 ± 1.40DE
Frankincense essential oil	0.5	2.00 ± 1.27H	0.00 ± 0.00E
Frankincense essential oil	0.2	6.20 ± 1.93GH	1.40 ± 1.40DE
*Eucalyptus* essential oil	2.0	15.40 ± 1.72EFG	2.80 ± 1.72CDE
*Eucalyptus* essential oil	0.5	7.60 ± 2.16GH	0.00 ± 0.00E
*Eucalyptus* essential oil	0.2	21.00 ± 10.58E	1.40 ± 1.40DE
Tea tree essential oil	2.0	18.60 ± 2.21EF	8.20 ± 3.25CDE
Tea tree essential oil	0.5	11.40 ± 2.09FGH	4.00 ± 2.63CDE
Tea tree essential oil	0.2	8.60 ± 1.75GH	2.60 ± 2.60CDE
Lavender essential oil	2.0	100.00 ± 0.00A	100.00 ± 0.00A
Lavender essential oil	0.5	100.00 ± 0.00A	100.00 ± 0.00A
Lavender essential oil	0.2	95.00 ± 5.00A	94.60 ± 5.40A
Citronella essential oil	2.0	100.00 ± 0.00A	100.00 ± 0.00A
Citronella essential oil	0.5	95.60 ± 4.40A	97.40 ± 2.60A
Citronella essential oil	0.2	100.00 ± 0.00A	100.00 ± 0.00A
Peppermint essential oil	2.0	100.00 ± 0.00A	100.00 ± 0.00A
Peppermint essential oil	0.5	100.00 ± 0.00A	100.00 ± 0.00A
Peppermint essential oil	0.2	5.40 ± 0.60H	10.60 ± 3.36C
Lemon essential oil	2.0	34.20 ± 2.60D	9.40 ± 3.36CD
Lemon essential oil	0.5	8.20 ± 0.86GH	0.00 ± 0.00E
Lemon essential oil	0.2	9.00 ± 0.707GH	1.40 ± 1.40DE
Rosemary essential oil	2.0	100.00 ± 0.00A	100.00 ± 0.00A
Rosemary essential oil	0.5	100.00 ± 0.00A	100.00 ± 0.00A
Rosemary essential oil	0.2	73.80 ± 2.56B	78.60 ± 7.67B

*Different capital letters in the same column in the table indicate a significant difference at *p* < 0.05.

### Repellent Activity Test Results on Armyworm Larvae

**FIGURE 1:**
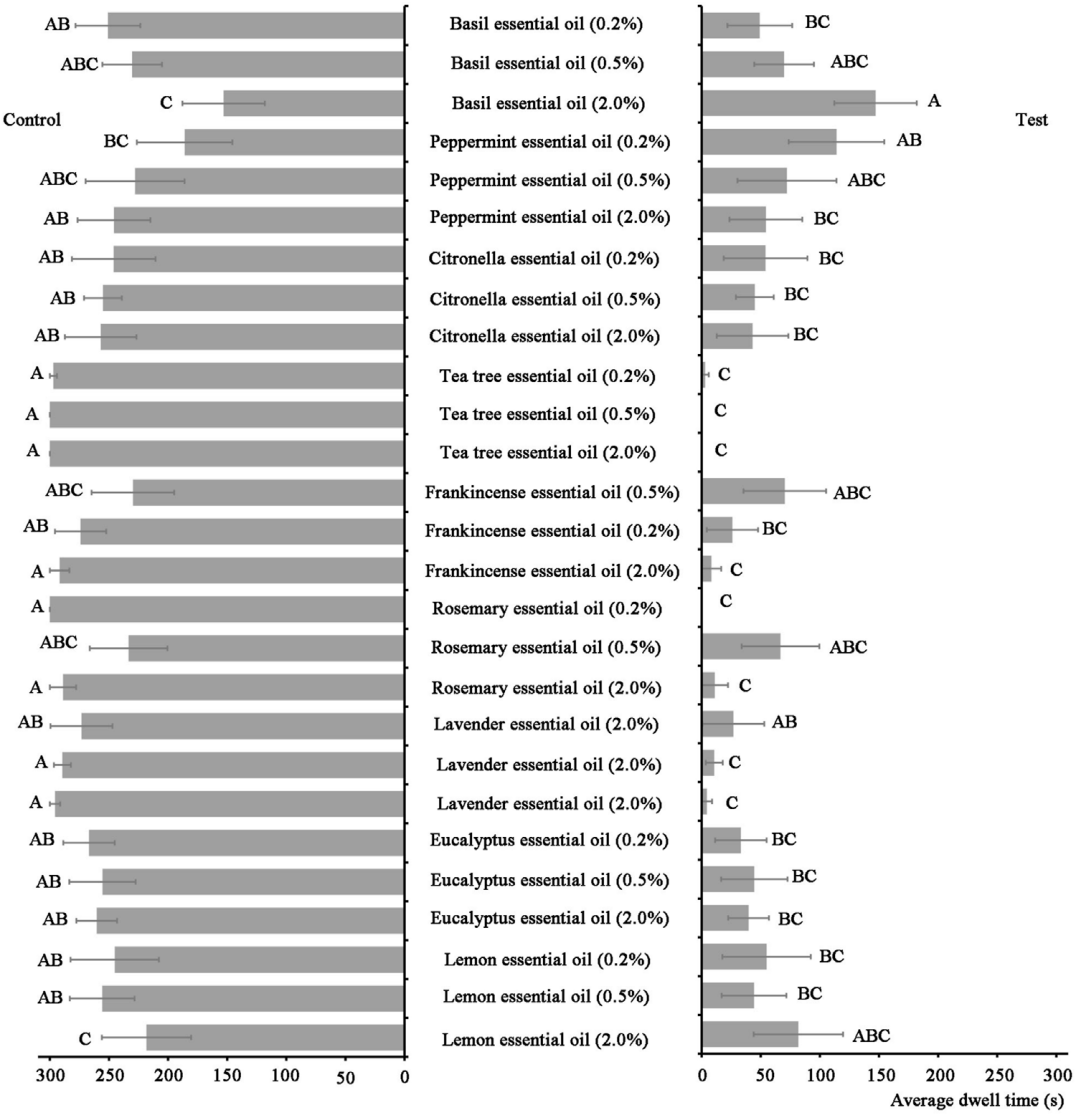
An average dwell time of armyworm larvae in different plant essential oils. Different capital letters in the figure indicate significant differences in the mean residence time of each concentration of plant essential oil treatment at *p* < 0.05.

Figure 1 showed that lavender essential oil, frankincense essential oil, and tea tree essential oil had obvious repellent effects on armyworm larvae. Among them, tea tree essential oil had an average dwell time of 0 s at the contents of 0.5 and 2.0%, meanwhile, rosemary essential oil had an average dwell time of 0 s at the content of 0.2%, indicating that tea tree essential oil and rosemary essential oil revealed the best repellent activity on armyworm larvae.

### Fumigation Activity Test Results on Armyworm Larvae

It can be seen from Table 2 that the fumigation activity on armyworm larvae has gradually increased with the treatment time. Among them, tea tree essential oil with a content of 2.0% had the best fumigation activity against armyworm larvae, the corrected mortality rates at 48, 72, 96, and 120 h were 26.33, 48.67, 73.33, and 86.67%, respectively, followed by rosemary essential oil with the content of 2.0%, its corrected mortality rates were 26.17, 28.67, 46.33, and 64.33% at 48, 72, 96, and 120 h, respectively.

**TABLE 2:** The fumigation activity of plant essential oils against armyworms larvae.

Plant essential oils	Content (%)	Corrected mortality rate (%)*			
		48 h	72 h	96 h	120 h
Basil essential oil	2.0	11.33 ± 8.09BCD	22.00 ± 5.86CD	33.33 ± 3.76C	46.67 ± 3.76C
Basil essential oil	0.5	11.00 ± 2.00BCD	13.33 ± 3.76DEF	17.67 ± 4.67DEG	26.67 ± 6.67FGHI
Basil essential oil	0.2	15.67 ± 8.09BCD	15.67 ± 8.09DEF	24.67 ± 2.33CDEF	22.41 ± 3.76FGHI
Frankincense essential oil	2.0	7.00 ± 0.00BCD	11.00 ± 2.00DEF	15.67 ± 4.33DEFG	20.00 ± 4.04GHIJ
Frankincense essential oil	0.5	2.33 ± 2.33D	4.67 ± 2.33F	4.67 ± 2.33H	15.33 ± 2.33HIJ
Frankincense essential oil	0.2	4.67 ± 2.33BCD	6.67 ± 3.76EF	11.00 ± 5.86EFGH	13.33 ± 3.76HIJ
*Eucalyptus* essential oil	2.0	13.33 ± 3.76ABCD	20.00 ± 0.00CDE	27.00 ± 0.00CD	42.33 ± 2.33CDE
*Eucalyptus* essential oil	0.5	6.67 ± 3.76BCD	11.33 ± 4.33DEF	15.33 ± 2.33DEFG	22.33 ± 2.33FGHI
*Eucalyptus* essential oil	0.2	7.00 ± 0.00BCD	11.00 ± 2.00DEF	24.33 ± 8.09GH	24.33 ± 8.09FGHI
Tea tree essential oil	2.0	26.33 ± 6.67A	48.67 ± 4.33A	73.33 ± 10.04A	86.67 ± 7.80A
Tea tree essential oil	0.5	13.33 ± 3.76ABCD	15.33 ± 2.33DEF	22.33 ± 2.33CDEF	44.67 ± 2.33C
Tea tree essential oil	0.2	9.00 ± 2.00BCD	13.33 ± 3.76DEF	13.33 ± 3.76DEFG	17.67 ± 2.33HIJ
Lavender essential oil	2.0	4.33 ± 4.33CD	9.00 ± 2.00DEF	13.33 ± 3.76DEFG	20.00 ± 4.04GHI
Lavender essential oil	0.5	4.67 ± 2.33BCD	7.00 ± 0.00DEF	7.00 ± 0.00GH	11.00 ± 2.00IJ
Lavender essential oil	0.2	7.00 ± 0.00BCD	7.00 ± 0.00DEF	11.00 ± 2.00EFGH	15.33 ± 2.33HIJ
Citronella essential oil	2.0	11.33 ± 4.33BCD	15.33 ± 2.33DEF	26.67 ± 3.76CD	37.67 ± 7.86CDEF
Citronella essential oil	0.5	7.00 ± 0.00 BCD	11.00 ± 2.00DEF	13.00 ± 0.00DEFG	24.33 ± 8.09FGHI
Citronella essential oil	0.2	18.00 ± 5.86ABC	20.00 ± 4.04CDE	20.00 ± 4.04CDEF	22.33 ± 2.33EFGH
Peppermint essential oil	2.0	9.00 ± 5.86BCD	17.67 ± 2.33CDE	24.33 ± 4.33CDE	29.00 ± 5.86DEFG
Peppermint essential oil	0.5	11.33 ± 4.33BCD	15.33 ± 2.33DEF	17.67 ± 2.33DEFG	22.33 ± 2.33FGHI
Peppermint essential oil	0.2	7.00 ± 0.00BCD	7.00 ± 0.00DEF	9.00 ± 2.00 FGH	11.00 ± 2.00CD
Lemon essential oil	2.0	13.33 ± 6.67ABCD	13.33 ± 6.67DEF	24.33 ± 4.33CDE	35.33 ± 2.33CDEF
Lemon essential oil	0.5	4.67 ± 2.33BCD	15.33 ± 2.33DEF	15.33 ± 2.33DEFG	20.00 ± 4.04GHIJ
Lemon essential oil	0.2	2.33 ± 2.33D	6.67 ± 3.76EF	6.67 ± 3.76GH	9.00 ± 5.86J
Rosemary essential oil	2.0	26.17 ± 7.80A	28.67 ± 8.09BC	46.33 ± 6.67B	64.33 ± 8.67B
Rosemary essential oil	0.5	20.00 ± 4.04AB	35.67 ± 5.93B	53.33 ± 3.76B	62.33 ± 7.86B
Rosemary essential oil	0.2	11.00 ± 5.86ABC	22.33 ± 2.33CD	26.67 ± 3.76CD	35.67 ± 4.33CDEF

*Different capital letters in the same column in the table indicate a significant difference at *p* < 0.05.

### Contact Activity Test Results on Armyworm Larvae

It can be seen from Table 3, that the contact activity on armyworm larvae has gradually increased with the treatment time. Among them, basil essential oil at the content of 2.0% revealed the best contact activity on armyworm larvae with the corrected mortality rates of 40.00, 48.67, and 57.67% at 48, 72, and 96 h, respectively. Lemon essential oil with a content of 2.0% had the best contact activity (66.67%) on armyworm larvae at 120 h.

**TABLE 3:** The contact activity of plant essential oils against armyworms larvae.

Plant essential oils	Content (%)	Corrected mortality rate (%)*			
		48 h	72 h	96 h	120 h
Basil essential oil	2.0	40.00 ± 4.04A	48.67 ± 4.33A	57.67 ± 9.60A	62.00 ± 5.86AB
Basil essential oil	0.5	7.00 ± 0.00EFG	17.67 ± 4.67EFGHI	26.67 ± 3.76EFIJ	35.33 ± 2.33EFGHI
Basil essential oil	0.2	17.67 ± 4.67CDEF	17.67 ± 4.67EFGHI	31.00 ± 5.86DEFGH	38.00 ± 5.86DEFGH
Frankincense essential oil	2.0	7.00 ± 0.00EFG	11.00 ± 2.00GHIJ	22.33 ± 2.33FGHIJ	29.00 ± 2.00GHIJK
Frankincense essential oil	0.5	2.33 ± 2.33G	6.67 ± 3.76IJ	8.67 ± 4.33JK	15.67 ± 8.09KLM
Frankincense essential oil	0.2	2.33 ± 2.33G	7.00 ± 0.00HIJ	13.33 ± 3.76IJK	13.33 ± 3.76LM
*Eucalyptus* essential oil	2.0	13.33 ± 7.80DEFG	24.33 ± 5.93CDEFG	44.67 ± 2.33BCD	64.67 ± 2.33A
*Eucalyptus* essential oil	0.5	6.67 ± 3.76EFG	20.00 ± 4.04EFGH	29.00 ± 5.86EFGH	49.00 ± 2.00BCDE
*Eucalyptus* essential oil	0.2	6.67 ± 2.33EFG	13.00 ± 0.00GHIJ	20.00 ± 0.00GHIJK	20.00 ± 0.00JKLM
Tea tree essential oil	2.0	15.67 ± 5.93CDEFG	22.33 ± 4.67DEFG	40.33 ± 6.67CDE	55.33 ± 2.33ABC
Tea tree essential oil	0.5	11.33 ± 4.33EFG	24.67 ± 2.33CDEF	33.33 ± 3.76CDEFG	49.00 ± 5.86BCDE
Tea tree essential oil	0.2	15.33 ± 2.33CDEFG	17.67 ± 2.33EFGHI	26.67 ± 3.76EFGHIJ	26.67 ± 3.76HIJKL
Lavender essential oil	2.0	2.33 ± 2.33G	6.67 ± 6.67IJ	13.33 ± 7.80IJK	15.67 ± 5.93KLM
Lavender essential oil	0.5	2.33 ± 2.33G	2.33 ± 2.33J	6.67 ± 3.76K	9.00 ± 2.00M
Lavender essential oil	0.2	6.67 ± 3.76EFG	13.00 ± 0.00GHIJ	20.00 ± 4.04HIJK	20.00 ± 4.04JKLM
Citronella essential oil	2.0	20.00 ± 0.00CDE	29.00 ± 2.00CDE	35.33 ± 2.33CDEF	42.33 ± 4.67CDEFG
Citronella essential oil	0.5	26.67 ± 6.67BC	33.33 ± 3.76CD	44.33 ± 4.33BCD	55.33 ± 2.33ABC
Citronella essential oil	0.2	24.67 ± 2.33BCD	26.67 ± 3.76CDE	40.00 ± 4.04CDE	51.00 ± 5.86BCD
Peppermint essential oil	2.0	9.00 ± 2.00EFG	17.67 ± 2.33EFGHI	29.00 ± 2.00EFGH	33.33 ± 3.76EFGHIJ
Peppermint essential oil	0.5	7.00 ± 0.00EFG	11.00 ± 2.00GHIJ	17.67 ± 2.33HIJK	22.33 ± 2.33IJKLM
Peppermint essential oil	0.2	6.67 ± 3.76EFG	6.67 ± 3.76IJ	13.33 ± 3.76IJK	22.00 ± 5.86IJKLM
Lemon essential oil	2.0	15.67 ± 5.93CDEFG	35.67 ± 5.93BC	47.00 ± 0.00ABC	66.67 ± 3.76A
Lemon essential oil	0.5	33.33 ± 3.76AB	46.67 ± 3.76AB	55.33 ± 2.33AB	64.67 ± 2.33A
Lemon essential oil	0.2	13.33 ± 3.76DEFG	22.33 ± 4.67DEFG	29.00 ± 2.00EFGH	37.67 ± 4.67DEFG
Rosemary essential oil	2.0	20.00 ± 4.04CDE	22.00 ± 5.86DEFG	33.00 ± 0.00DEFG	42.33 ± 2.33DEFG
Rosemary essential oil	0.5	18.00 ± 5.86CDE	24.67 ± 2.33CDEF	40.00 ± 4.04CDE	49.00 ± 5.86BCDE
Rosemary essential oil	0.2	8.67 ± 4.33EFG	22.00 ± 5.86DEFG	33.00 ± 0.00DEFG	44.67 ± 2.33CDEF

*Different capital letters in the same column in the table indicate a significant difference at *p* < 0.05.

### Synergistic Effect of Plant Essential Oils and Indoxacarb on Armyworm Larvae

As can be seen from Table 4, different mixture ratios of plant essential oil and indoxacarb with different synergistic effects on armyworm larvae. Among them, the combination of citronella essential oil and indoxacarb with the ratio of 5:1 had the best synergistic effect on armyworm larvae at 96 h, and the synergistic ratio was reached 100.00%, indicating that the plant essential oil could significantly improve the mortality rate of indoxacarb on armyworm larvae.

**TABLE 4:** Synergistic effects of different plant essential oils and indoxacarb.

Combination	Mixture ratio	Synergistic ratio (%)*			
		24 h	48 h	72 h	96 h
Basil essential oil:indoxacarb	5:1	50.00 ± 5.77BCD	53.33 ± 3.33CDEF	66.67 ± 8.82ABCDE	70.00 ± 5.77CDEF
Basil essential oil:indoxacarb	10:1	50.00 ± 5.77BCD	56.67 ± 3.33BCDEF	76.67 ± 12.02ABCD	76.67 ± 12.02ABCD
Basil essential oil:indoxacarb	20:1	13.33 ± 3.33FG	23.33 ± 3.33H	46.67 ± 8.82DEF	53.33 ± 12.02EF
Frankincense essential oil:indoxacarb	5:1	30.00 ± 5.77DEFG	36.67 ± 6.67FGH	45.00 ± 20.21DEF	76.67 ± 3.33ABCD
Frankincense essential oil:indoxacarb	10:1	53.33 ± 6.67ABC	70.00 ± 5.77ABC	70.00 ± 5.77ABCDE	70.00 ± 5.77CDEF
Frankincense essential oil:indoxacarb	20:1	26.67 ± 8.82DEFG	46.67 ± 3.33DEFG	56.67 ± 3.33BCDEF	63.33 ± 3.33DEF
*Eucalyptus* essential oil:indoxacarb	5:1	56.67 ± 3.33AB	76.67 ± 3.33ABC	90.00 ± 5.77A	90.00 ± 5.77ABC
*Eucalyptus* essential oil:indoxacarb	10:1	50.00 ± 5.77BCD	56.67 ± 8.82BCDEF	80.00 ± 5.77ABC	96.67 ± 3.33AB
*Eucalyptus* essential oil:indoxacarb	20:1	16.67 ± 8.82FG	46.67 ± 8.82DEFG	66.67 ± 12.02ABCDE	90.00 ± 5.77ABC
Tea tree essential oil:indoxacarb	5:1	30.00 ± 5.77DEFG	43.33 ± 8.82EFGH	50.00 ± 5.77CDEF	50.00 ± 5.77F
Tea tree essential oil:indoxacarb	10:1	16.67 ± 3.33FG	36.67 ± 3.33FGH	43.33 ± 3.33EF	53.33 ± 3.33EF
Tea tree essential oil:indoxacarb	20:1	10.00 ± 5.77G	30.00 ± 5.77GH	33.33 ± 6.67F	63.33 ± 8.82DEF
Lavender essential oil:indoxacarb	5:1	56.67 ± 8.82AB	73.33 ± 6.67ABC	90.00 ± 10.00A	96.67 ± 3.33AB
Lavender essential oil:indoxacarb	10:1	56.67 ± 6.67AB	80.00 ± 5.77AB	83.33 ± 8.82AB	90.00 ± 5.77ABC
Lavender essential oil:indoxacarb	20:1	23.33 ± 14.53EFG	60.00 ± 5.77BCDE	66.67 ± 8.82ABCDE	80.00 ± 5.77ABCD
Citronella essential oil:indoxacarb	5:1	46.67 ± 8.82BCDE	76.67 ± 3.33ABC	93.33 ± 3.33A	100.00 ± 0.00A
Citronella essential oil:indoxacarb	10:1	50.00 ± 11.55BCD	66.67 ± 14.53ABCD	73.33 ± 14.53ABCDE	83.33 ± 12.02ABCD
Citronella essential oil:indoxacarb	20:1	26.67 ± 8.82DEFG	40.00 ± 10.00EFGH	73.33 ± 14.53ABCDE	80.00 ± 10.00ABCD
Peppermint essential oil:indoxacarb	5:1	50.00 ± 5.77BCD	76.67 ± 3.33ABC	83.33 ± 6.67AB	86.67 ± 8.82ABCD
Peppermint essential oil:indoxacarb	10:1	26.67 ± 8.82DEFG	40.00 ± 10.00EFGH	46.67 ± 14.53DEF	66.67 ± 8.82CDEF
Peppermint essential oil:indoxacarb	20:1	30.00 ± 5.77DEFG	40.00 ± 5.77EFGH	46.67 ± 6.67DEF	50.00 ± 5.77F
Lemon essential oil:indoxacarb	5:1	60.00 ± 5.77AB	80.00 ± 5.77AB	90.00 ± 5.77A	96.67 ± 3.33AB
Lemon essential oil	10:1	56.67 ± 3.33AB	73.33 ± 3.33ABC	76.67 ± 3.33ABCD	80.00 ± 0.00ABCD
Lemon essential oil:indoxacarb	20:1	56.67 ± 8.82AB	70.00 ± 10.00ABC	73.33 ± 8.82ABCDE	73.33 ± 8.82BCDE
Rosemary essential oil:indoxacarb	5:1	76.67 ± 6.67A	86.67 ± 3.33A	93.33 ± 3.33A	96.67 ± 3.33AB
Rosemary essential oil:indoxacarb	10:1	50.00 ± 5.77BCD	63.33 ± 8.82ABCDE	76.67 ± 12.02ABCD	83.33 ± 8.82ABCD
Rosemary essential oil:indoxacarb	20:1	36.67 ± 3.33BCDEF	63.33 ± 8.82ABCDE	76.67 ± 6.67ABCD	80.00 ± 5.77ABCD
indoxacarb	-	20.00 ± 5.77C	33.33 ± 3.33B	43.33 ± 3.33AB	43.33 ± 3.33AB

*Different capital letters in the same column in the table indicate a significant difference at *p* < 0.05.

## Discussion

Botanical pesticides play an increasingly important role in controlling agricultural pests. The effects of essential oils on the behavior, survival, and reproduction of various pests have been extensively studied (Mossa, 2016; Pavela and benelli, 2016; Sousa et al., 2021). At present, essential oils are considered as ideal potential green pesticides and have been well applied and recognized in forest pest control (Cetin et al., 2010). In this study, we demonstrated that nine plant essential oils, including basil essential oil, peppermint essential oil, lemon essential oil, rosemary essential oil, citronella essential oil, frankincense essential oil, eucalyptus essential oil, tea tree essential oil, and lavender essential oil, had significant biological activity against armyworm larvae. Among them, lavender essential oil and citronella essential oil had the best antifeedant effects on armyworm larvae, and the antifeedant rates in the three gradients of 2, 0.5, and 0.2% are all 100%. Zhang Y. et al. (2019) found that citronella essential oil and lemon essential oil with a concentration of 10 μL/ml had a higher antifeedant rate in a 2-day-old diamondback moth, and the antifeedant rate of calamus essential oil was 100% with the increase of concentration. Kosti´c et al. (2013) also found that water chestnut essential oil and nutmeg essential oil had strong antifeedant to gypsy moth, and when the concentration reached 0.1%, water chestnut essential oil doubled the antifeedant rate of gypsy moth larvae. Skuhrovec et al. (2020) suggested that essential oil concentrations had a significant effect on the feeding of the potato beetle *Leptinotarsa decemlineata*. Lavender and tea tree essential oils also had a significant avoidance effect on citrus psyllid adults and could inhibit the attraction of citrus leaves to psyllids (Mann et al., 2012). To sum up, it showed that the concentration of essential oil had a great influence on the feeding behavior of insects, and its antifeedant rate could increase with the increase of the concentration of essential oil within a certain concentration range. Therefore, it is necessary to add more concentration gradients of essential oils to further probe the effect of essential oil concentration on insect feeding. In addition, tea tree oil had a strong attracting effect on tobacco beetle [*Lasioderma serricorne* (Fabricius)] adults (Ren et al., 2022), but the attractive effect of tea tree oil on armyworm larvae in this study was extremely poor at two gradients of 2 and 0.5%.

In this study, nine plant essential oils showed significant avoidance activity against armyworm larvae. However, there were differences in the avoidance rate of armyworm larvae for each essential oil, which may be related to the volatile chemicals of plant essential oils. María et al. (2021) found that citronellic acid in citronella essential oil had a significant repelling effect on bed bugs, while menthone in peppermint essential oil had no apparent avoidance response to bed bugs. However, in this study, the three concentrations of peppermint essential oil produced strong avoidance activity on 4-days-old armyworm larvae, indicating that the volatile substances with repelling effect on armyworm larvae were the other main components. Kady Rocha et al. (2015) found that the main components of fennel essential oil respond differently to the behavior of *Aedes aegypti*. Taken together, it is shown that the behavioral responses of essential oils to insects are closely related to the main components of essential oils. For example, Sombra et al. (2020) believed that the behavioral changes of *Spodoptera frugiperda* caused by citronella essential oil were the effect of the main compound menthone (55.97%) and a few compounds such as geraniol (3.44%). Deletre et al. (2016) found that the main compound of citronella essential oil may react with insect olfactory receptors, leading to insect avoidance behavior. Santos et al. (2016) found that rosemary essential oil contained carvacrol (30.37%), α-himalayanene (10.38%), terpinene (7.96%), α-pinene (5.08%), and β-myrcene (3.90%), and other substances can cause symptoms such as hyperactivity, tremor, nerve paralysis, and muscle fatigue in caterpillars, resulting in a better insecticidal activity. El-wakeil, (2003) reported that carvacrol in essential oil had great insecticidal potential, which can compete with nicotinic acetylcholine receptors and can be used as an acetylcholinesterase inhibitor.

Basil essential oil, tea tree essential oil, lemon essential oil, and rosemary essential oil all showed high biological activity against 4-days-old armyworms in this experiment. It is more significant in combination with pesticides (indoxacarb), and the synergistic ratio of some essential oils reaches 100%. Yu et al. (2018) also found that the synergistic ratios of basil and citronella + lemon essential oil to pesticide (butene fipronil) were 1.3 and 1.5, respectively, which showed that essential oils had a significant synergistic effect on pesticides. Meanwhile, some studies had showed that the synergistic effect of essential oils could significantly affect the biological activity of pests (Pavela, 2014; Aungtikun et al., 2021). In addition, studies had showed that the effects of essential oils on insect bioactivity were related to insect species, treatment methods, and treatment time (Pavela et al., 2016; Giovanni et al., 2018; Lazarevi et al., 2020).

## Conclusion

In conclusion, 9 kinds of plant essential oils were used to explore the antifeedant activity, repellent activity, fumigation activity, contact activity, and synergistic effects on armyworms larvae. Our results showed that lavender and citronella essential oils had the greatest impact on the antifeedant activity (100.00%) on armyworm larvae at the contents of 2.0, 0.5, and 0.2%. Meanwhile, rosemary essential oil revealed the best repellent activity (average dwell time: 0 s) on armyworm larvae at the content of 0.2%. Moreover, tea tree essential oil and lemon essential oil had the best fumigation activity (86.67%) and contact activity (66.67%) against armyworm larvae at a content of 2.0%. In addition, the combination of citronella essential oil and indoxacarb with the ratio of 5:1 had the best synergistic effect (100.00%) on armyworm larvae at 96 h. Our results provide a reference for enriching the green control technology of armyworms larvae.

## Data Availability

The raw data supporting the conclusions of this article will be made available by the authors, without undue reservation.
